# Acknowledgement to the reviewers of GMS Journal for Medical Education

**DOI:** 10.3205/zma001522

**Published:** 2022-02-15

**Authors:** Martin R. Fischer, Götz Fabry

**Affiliations:** 1 GMS Journal for Medical Education, Chief Editor, Erlangen, Germany; 2 University Hospital, LMU Munich, Institute for Medical Education, Munich, Germany; 3 Albert-Ludwig-Universität Freiburg, Medizinische Fakultät, Abteilung für Medizinische Psychologie und Soziologie, Freiburg, Germany

## Acknowledgement

We gratefully acknowledge the valuable contribution of the following peer reviewers, who supported our Journal in 2021. We are looking forward to continuing our collaboration!

## Reviewers in alphabetical order

- Tigistu Adamu, Baltimore (USA)
- Olaf Ahlers, Berlin
- Obada al Halabi, Heidelberg
- Attila Altiner, Rostock
- Tsedeke Asaminew, Jimma (ETH)
- Birgit Babitsch, Osnabrück
- Ivan Bank, Amsterdam (NL)
- Anne Barzel, Ulm
- Daniel Bauer, Bern (CH)
- Erika Baum, Biebertal
- Andrea Baumann, Tübingen
- Ronja Behrend, Berlin
- Marianne Behrends, Hannover
- Katrin Bekes, Wien (A)
- Alexander Benz, München
- Pascal Berberat, München
- Joana Berger, Bern (CH)
- Markus Berndt, München
- Karin Binder, Regensburg
- Tanja Birrenbach, Bern (CH)
- Eva Maria Bitzer, Freiburg
- Jutta Bleidorn, Jena
- Klaus Böhme, Bochum
- Markus Borchelt, Berlin
- Stefan Bösner, Marburg
- Hans Martin Bosse, Düsseldorf
- Barbara Braun, Mannheim
- Jan Breckwoldt, Zürich (CH)
- Andreas Burger, Bochum
- Rainer Büscher, Essen
- Nora Celebi, Waiblingen
- Jean-Francois Chenot, Greifswald
- Irmgard Claßen-Linke, Aachen
- Monika Daubländer, Mainz
- Antje Degel, Berlin
- Martin A. Denvir, Edinburgh (UK)
- Konstantin Dimitriadis, München
- Andreas Dinkel, München
- Nadine Dreimüller, Mainz
- Nurith Epstein, München
- Gundula Ernst, Hannover
- Götz Fabry, Freiburg
- Nora Faubl, Pécs (HUN)
- Folkert Fehr, Sinsheim an der Elsenz
- Sabine Ingrid Fischbeck, Mainz
- Martin R. Fischer, München
- Volkhard Fischer, Hannover
- Johannes Forster, Merzhausen
- Julia Freytag, Berlin
- Hendrik Friederichs, Bielefeld
- Annette Fröhmel, Düsseldorf
- Martin Gartmeier, München
- Kirsten Gehlhar, Oldenburg
- Sabine Gehrke-Beck, Berlin
- Waltraud Georg, Berlin
- Marianne Giesler, Freiburg
- Heidrun Golla, Köln
- Maryna Gornostayeva, Mainz
- Heide Götze, Leipzig
- Joachim Graf, Tübingen
- Matthäus Grasl, Wien (A)
- Julia Grundnig, Wien (A)
- Markus Gulich, Ulm
- Stefan Gysin, Luzern (CH)
- Matthias Haase, Magdeburg
- Eckhart G. Hahn, Erlangen
- Christian Hammer, Erlangen
- Marietta Handgraaf, Bochum
- Inga Hege, Augsburg
- Nicole Heitzmann, München
- Linn Hempel, Halle/Saale
- Doreen Herinek, Berlin
- Anne Herrmann-Werner, Tübingen
- Martina Heßbrügge, Essen
- Monika Himmelbauer, Wien (A)
- Barbara Hinding, Mainz
- Anke Hollinderbäumer, Mainz
- Christopher Holzmann-Littig, München
- David Hörburger, St. Gallen (CH)
- Sören Huwendiek, Bern (CH)
- Daniel Ithaler, Graz (A)
- Nana Jedlicska, München
- Ralf Jendyk, Münster
- Canar Kamisli, Bochum
- Yassin Karay, Köln
- Andreas Klement, Halle/Saale
- Katja Koelkebeck, Essen
- Karin Kraft, Rostock
- Sandy Kujumdshiev, Leipzig
- Raphael Kunisch, Erlangen
- Evelyn Lamy, Freiburg
- Henriette Löffler-Stastka, Wien (A)
- Gabriele Lutz, Witten
- Cornelia Mahler, Tübingen
- Jörg Marienhagen, Augsburg
- Gabriella Marx, Hamburg
- Gregor Massoth, Bonn
- Jan Matthes, Köln
- Stefanie Merse, Essen
- Annette Mettler, Bern (CH)
- Janet Michel, Basel (CH)
- Parisa Moll-Khosrawi, Hamburg
- Andreas Möltner, Heidelberg
- Sören Moritz, Köln
- Brigitte Müller-Hilke, Rostock
- Ede Nagy, Heidelberg
- Marcus Neudert, Dresden
- Dino Carl Novak, Berlin
- Thomas Nowak, Mainz
- Falk R. Ochsendorf, Frankfurt/Main
- Wolfgang Öchsner, Ulm
- Stefanie Otten-Marré, Düsseldorf
- Volker Paulmann, Hannover
- Dorothea Penders, Berlin
- Anthea Peters, Bonn
- Bettina Pfleiderer, Münster
- Severin Pinilla, Bern (CH)
- André Posenau, Bochum
- Alexander Rahman, Hannover
- Peter Riegler, Wolfenbüttel
- Bernd Romeike, Rostock
- Caroline Rometsch-Ogioun El Sount, Tübingen
- Marco Roos, Erlangen
- Jerome Rotgans, Witten
- Thomas Rotthoff, Augsburg
- LindaS anftenberg, München
- Stefan Schauber, Oslo (NO)
- Jörg Schelling, Martinsried
- Ralf Schmidmaier, München
- Anita Schmidt, Erlangen
- Eva Schönefeld, Münster
- Teresa Schreckenbach, Frankfurt/Main
- Jeannine Schübel, Dresden
- Katrin Schüttpelz-Brauns, Mannheim
- Thomas Shiozawa-Bayer, Tübingen
- Anne Simmenroth, Würzburg
- Angelika Simonsohn, München
- Andreas Söhnel, Greifswald
- Beat Sottas, Bourguillon (CH)
- Jasmina Sterz, Frankfurt/Main
- Tina Stibane, Marburg
- Beate Stock-Schröer, Witten
- Dietrich Stoevesandt, Halle/Saale
- Christoph Stosch, Köln
- Irmgard Streitlein-Böhme, Bochum
- Fabian Stroben, Berlin
- Cynthia Szalai, Mülheim a.d. Ruhr
- Sefik Tagay, Köln
- Muhammad Tariq, Nairobi (KEN)
- Nils Thiessen, Bielefeld
- Christian Thrien, Köln
- Daniel Tolks, Lüneburg/Bielefeld
- Monica van de Ridder, Michigan (USA)
- Barbara Vogel, Berlin
- Thomas von Lengerke, Hannover
- Michaela Wagner-Menghin, Wien (A)
- Alexander Waschkau, Lübeck
- Marc Weidenbusch, München
- Mario Wijnen-Meijer, München
- Barbara Woestmann, Bochum
- Solomon Worku, Addis Ababa (ETH)
- Anja Zimmermann, Berlin
- Jan Zottmann, München

